# Pet dogs (*Canis familiaris*) re-engage humans after joint activity

**DOI:** 10.1007/s10071-023-01774-1

**Published:** 2023-04-13

**Authors:** Molly Byrne, Daniel J. Horschler, Mark Schmitt, Angie M. Johnston

**Affiliations:** 1 Department of Psychology, Boston College, 140 Commonwealth Ave., Chestnut Hill, MA 02467 USA; 2 Department of Psychology, Yale University, 2 Hillhouse Ave, New Haven, CT 06511 USA

**Keywords:** Shared intentionality, Theory of mind, Canine cognition, Social cognition, Cooperation

## Abstract

Joint intentionality, the mutual understanding of shared goals or actions to partake in a common task, is considered an essential building block of theory of mind in humans. Domesticated dogs are unusually adept at comprehending human social cues and cooperating with humans, making it possible that they possess behavioral signatures of joint intentionality in interactions with humans. Horschler and colleagues (Anim Behav 183: 159–168, 2022) examined joint intentionality in a service dog population, finding that upon interruption of a joint experience, dogs preferentially re-engaged their former partner over a passive bystander, a behavior argued to be a signature of joint intentionality in human children. In the current study, we aimed to replicate and extend these results in pet dogs. One familiar person played with the dog and then abruptly stopped. We examined if dogs would preferentially re-engage the player instead of a familiar bystander who was also present. Consistent with the findings of Horschler and colleagues (Anim Behav 183: 159–168, 2022), pet dogs preferentially gazed toward and offered the toy to the player significantly more than the familiar bystander. However, no difference was observed in physical contact. These findings provide preliminary evidence for behavioral signatures of joint intentionality in pet dogs, but future work is needed to understand whether this phenomenon extends to other contexts.

**Supplementary Information:**

The online version contains supplementary material available at 10.1007/s10071-023-01774-1.

## Introduction

Joint intentionality is an early component of theory of mind that requires an understanding of others’ goals and a coordination of intentions to participate in a shared goal together (Searle 1979; Tomasello and Carpenter 2007; Tomasello et al. 2005; Tomasello and Moll 2010; Tuomela 2002; Tuomela and Miller 1988). Scholars have proposed that joint intentionality is a building block of tracking others’ intentions, and that this skill is requisite of more advanced theories of others’ minds (Tomasello 2018). Past work has assessed joint intentionality through tests of joint commitment to an activity (Gräfenhain et al. 2009; *in apes:* MacLean and Hare 2013). To share intentions, each actor must commit to a common goal in an activity and understand that their partner is also committed to accomplishing that goal together (Tomasello et al. 2005). Therefore, if one actor ceases to participate, the goal cannot be accomplished, because togetherness is integral to the shared goal (Tomasello and Carpenter 2007). Most research has suggested that joint intentionality is unique to humans (Buttelmann 2022; Engelmann and Tomasello 2018; Tomasello, et al. 2005; Warneken et al. 2006); however, some researchers have suggested that play may be an ecologically relevant correlate to joint commitment in animals (Heesen et al. 2017, 2021).

However, examining the abilities of other social animals allows a window into the evolution of cognitive abilities such as joint intentionality. For example, the domestic dog has adapted to the human social world and exhibits many signs of understanding humans’ cooperative signals (MacLean et al. 2017; Miklosi and Topal 2013). Dogs’ roles in the human social world have led to heightened cooperation and environments in which rudimentary forms of joint intentionality may have emerged. Dogs live in close proximity to humans, and through the process of domestication have developed an unusual tolerance for and sensitivity to humans (Ben-Aderet et al. 2017; Bray et al. 2020, 2021; Duranton et al. 2017; Hare et al. 2005; Kaminski et al. 2011; Lakatos et al. 2009; Salomons et al. 2021; Teglas et al. 2012; Topal et al. 2009). Moreover, there is preliminary evidence that dogs may be sensitive to humans’ goals and intentions (Marshall-Pescini et al. 2013, 2014; Passalacqua et al. 2011; Piotti and Kaminski 2016; Schünemann et al. 2021). Together, these findings indicate that dogs may have evolved specialized mechanisms to interact with the human social world. Given that the evolutionary path of dogs has resulted in many social abilities previously thought to be unique to the humans (Johnston et al. 2017), we examined whether dogs have the potential to exhibit joint intentionality with humans.

In our previous work, we designed a task based on social play to test if dogs show behavioral signatures of joint intentionality in interactions with humans by demonstrating joint commitment (Horschler et al. 2022). In this task, one person (the ‘player’) engaged the dog in play for 45 s, while a second person (the ‘bystander’) watched, and then both people sat passively for 30 s. Previous work with children and chimpanzees has suggested that re-engagement behaviors (e.g., offering objects involved in play) are one behavioral signature of joint intentionality (Warneken et al. 2006; Warneken and Tomasello 2007). We found that dogs preferentially attempted to re-engage the player over the bystander by looking at, touching, and offering toys to the player significantly more than the bystander. This suggests that dogs may have formed a shared goal with the player to participate in the social game. However, this experiment was conducted exclusively with service-dogs-in-training, including only Labrador retrievers, golden retrievers, and Labrador x golden retriever crosses, all around 2 years of age. In the current study, we tested whether these findings extend to a more diverse sample of pet dogs. If dogs form shared experiences with humans, we expected them to show more looking, more physical contact, more toy offering, and more vocalizations toward the player than the bystander during the interruption period.

## Methods

This study was pre-registered at https://aspredicted.org/YLL_LQX before any data collection began.

### Participants

To sample a broad range of dogs, we recruited 36 pet dogs from a wide variety of breeds and ages (see Table S1 in the supplementary materials for demographics). All dogs were tested in one session in their own homes via Zoom, with two humans familiar to the dogs acting as the experimenters. The study was conducted in accordance with Boston College’s IACUC approval of ethically conducted animal studies, and the Boston College’s IRB approval of ethically collected human video data.

### Exclusions

As described in our pre-registration, we excluded trials where the citizen scientists made errors or did not follow directions (20), where the citizen scientists talked or tried to pet the dog in the re-engagement phase (3), and where the dog was not visible on the video for more than half of the re-engagement phase (4). One dog had all four trials excluded for the above reasons. Two additional dogs were excluded entirely (all four trials), one due to a video recording error and one due to the dog being unmanageable. In total, 109 trials from 33 dogs were included in the final analysis.

### Set up

Citizen scientists were called by one of our researchers using Zoom and asked to select a preferred toy for the study. Our researcher then explained and practiced the procedure with the citizen scientists. All video was captured via Zoom recording. Video consent was obtained for all dogs and humans in the experiment.

### Procedure

Our procedure was identical to that of our previous research with service dogs (Horschler et al. 2022) with one exception: because citizen scientists in the current study were both familiar with the dog, they rotated roles, so each dog had two trials of play with each person. Both people were familiar to the dog, but any differences in the dog’s behavior toward the two people would not be confounded with who was the player and who was the bystander, since each person had two trials in each role.

**Fig. 1:**
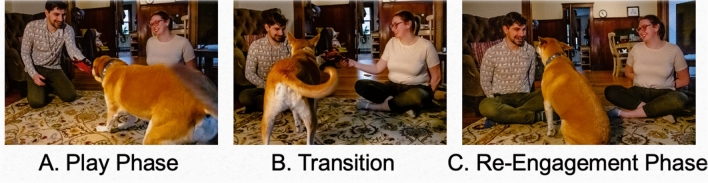
An example of what the image would look like in the duration of the experiment

In each trial, one citizen scientist served as the player, and the other as the bystander; both started seated on the floor arms-length apart from one another. Each trial consisted of a play phase, transition, and re-engagement phase (see Fig. 1). In the play phase, the player played with the dog using the pre-selected toy for 45 s; however, the dog preferred, including talking, while the bystander sat silently with hands behind their back. The player was asked to try to remain in front of the camera as much as possible. During the subsequent transition, the player sat in their original position, the bystander and player held the toy together, called the dog by name, and dropped the toy. This transition ensured that both citizen scientists had possession of the toy and had spoken to the dog equally recently. Finally, in the re-engagement phase, both people sat quietly with their hands behind their backs for 30 s, smiling and nodding if the dog made eye contact with them. Each dog participated in four trials, and each person took turns being the player and the bystander, such that both occupied each role two times, alternating roles. Trials immediately followed one another whenever the citizen scientists and dogs were ready to continue.

### Coding

We coded all measures with BORIS software. The third author (M.S.) and one naïve analyst reliability coded behaviors from the re-engagement phase of all trials. Reliability was determined by a correlation of both coders’ ratings.

Looking behavior (reliability: *r* = 0.96) was a continuous variable, defined as time spent with the dog’s nose or eyes directed above either person’s shoulders at their face. Physical contact (*r* = 0.88) was a continuous variable defined as time spent with any part of the dog physically touching any part of either person. Toy offering (*r* = 0.86) was defined as dropping the toy within arm’s length of either person, and was a count variable of total number of offers. Vocalizations were defined as any sounds the dog made while looking at the person, and were a count variable of total number of vocalizations. Reliability was only moderate for vocalizations (*r* = 0.60), but because vocalizations occurred on less than 10% of trials, this measure was not included in further analyses.

### Analysis

For our main analyses, we fit three linear mixed models, one for each of our pre-registered outcome variables: looking (identity link function and Gaussian error distribution), physical contact (identity link function and Gaussian error distribution), and toy offering (log-link function and Poisson error distribution). In each model, the person’s role (player or bystander) was included as a predictor, and the dog’s identity as a random effect. We fit all models in R version 4.1.1 and assessed the effects of each term using the ‘Anova’ function from the ‘car’ package to produce analysis of deviance tables using Type II Wald chi-square tests for model comparisons (Fox and Weisberg 2019).

As exploratory analyses, we also ran one sample t tests after transforming our continuous outcome measures (looking and physical contact) into difference scores (player-directed behavior divided by the sum of player-directed behavior and bystander-directed behavior), because player- and bystander-directed behaviors are not fully independent of each other (e.g., if a dog is looking at the player, they cannot also be looking at the bystander during that time). The results of these analyses mirrored those from our main analyses presented below, and are therefore presented in the Supplementary Materials. We also fit models including trial number as a predictor. In none of these models did trial number have a significant effect, indicating that behavior did not significantly change across trials, so they are also presented in the Supplementary Materials.

## Results

**Fig. 2:**
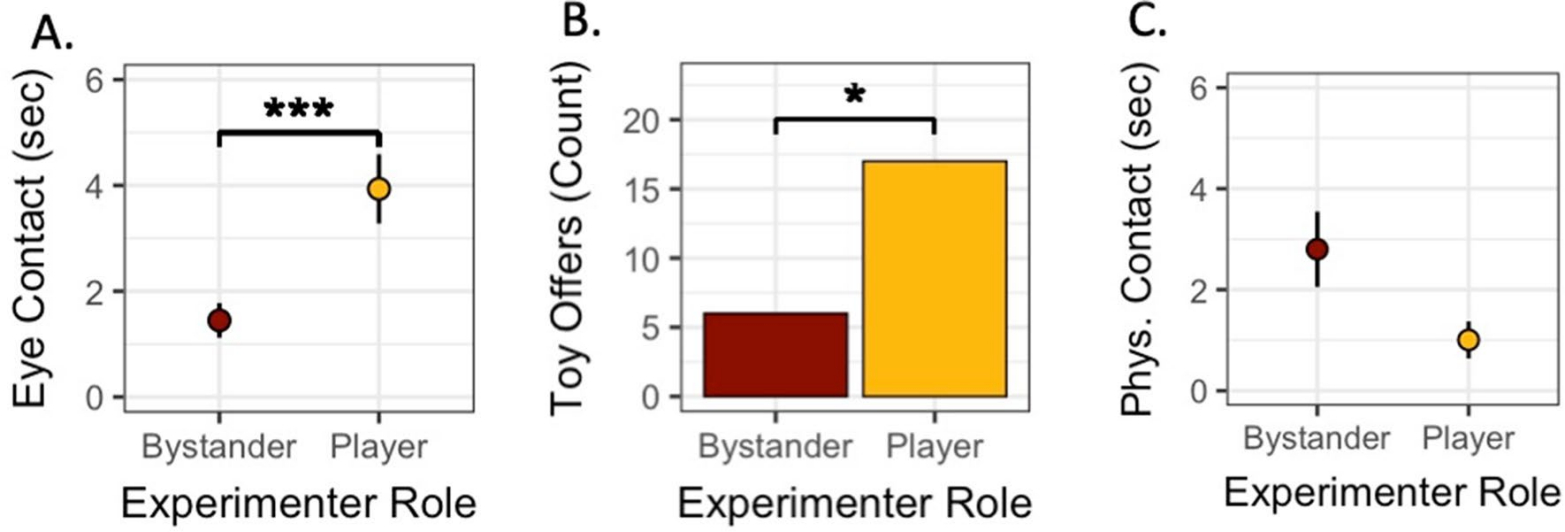
**A** Dogs made significantly more eye contact with the player than the bystander (mean eye contact in seconds; error bars represent standard error). **B** Dogs offered the toy significantly more to the player than the bystander (total count of offers to player and to bystander). **C** There was no significant difference between player-directed and bystander-directed physical contact (mean seconds of physical contact; error bars represent standard error)

We found a significant effect of the person’s role on looking behavior (see Fig. 2A), where dogs looked longer at the player (mean ± SD = 3.93 ± 6.85) than the bystander (1.45 ± 3.43; *χ*^2^(1) = 16.95, *p* < 0.001) with a medium-effect size (*d* = 0.56). We also found a significant effect of the person’s role on toy offering (see Fig. 2B), where dogs offered the toy more frequently to the player (17 occurrences) than the bystander (6 occurrences; *χ*^2^(1) = 4.82, *p* = 0.028) with a large effect size (*d* = 1.04). While these results are consistent with the results found by Horschler and colleagues (2022), unlike in that study, we did not find a significant effect of the person’s role on the amount of time dogs spent in physical contact with either person (see Fig. 2C; Player: 1.01 ± 3.82; Bystander: 2.8 ± 7.8; *χ*^2^(1) = 3.66, *p* = 0.056).

## Discussion

In line with our previous work (Horschler et al. 2022), we found that pet dogs preferentially attempted to re-engage their former partner in joint social play over a passive bystander. Upon interruption, they looked significantly longer at the player than the bystander, and also offered the toy significantly more to the player than the bystander. This supports the idea that pet dogs show some behavioral signatures of joint intentionality with humans.

However, in our previous work (Horschler et al. 2022), we not only found significantly greater player-directed looking and toy offering, but also physical contact. Our lack of significant findings with respect to physical contact may have resulted from the familiarity of the dogs with the citizen scientists, as both experimenters (citizen scientists) in the current study were highly familiar to the dogs rather than being strangers as in Horschler and colleagues (2022). Given that dogs experience greater stress reduction when pet by their owner as compared to a stranger (Kuhne et al. 2014), we might expect that dogs would not differentiate as strongly between two highly familiar people when seeking physical contact. Additionally, due to our definition of physical contact, dogs were said to be in physical contact even when not looking at either person. Therefore, physical contact may not be as strong of an index of re-engagement as our other measures, and instead may be more indicative of seeking comfort or general emotional regulation.

The results of the current study extend the results of our earlier work in Horschler and colleagues (2022) with a new population. Our study suggests that behavioral signatures of joint intentionality in dogs are not due to high levels of specialized training, not indicative of a trait bred specifically into a line of working dogs, and not exclusively characteristic of retrievers. Instead, the current study provides evidence that the expression of re-engagement behaviors (looking and toy-dropping preferentially toward a previous partner) is generalizable to a broader population of human-socialized dogs.

Our study shows evidence that even household dogs without specific training or breeding show some behavioral signatures of joint intentionality. However, more work is needed to understand how far this extends. While dogs may have some rudimentary skills that form the building blocks of joint intentionality, we cannot conclude that they are capable of joint intentionality from this method alone. However, because our design includes both the bystander and the player, if dogs were simply using humans as social tools or exhibiting a preference for interaction, they would not exhibit a preference for re-engaging the player, but instead direct these behaviors uniformly to any present human. It could be argued that dogs are merely using associative learning to engage the person whom they consider most likely to play in the future. However, in our design, we included the transition phase to make sure that both people held the toy and called the dog’s name immediately before the re-engagement phase began, so dogs could not simply choose the last person who called their attention or touched the toy. We also counterbalanced which citizen scientist was the player and the bystander across trials, so dogs could not build an association with one person simply being more likely to play. Had dogs simply drawn the association that one person was more likely to play, there would not have been differences between the player and bystander across trials. Even so, we cannot determine with certainty why dogs appear to discriminate between the player and the bystander.

There are several limitations to this study. First, our use of the transition phase could be interpreted as an invitation for engagement, and thus the re-engagement behaviors we observed may be in response to a perceived solicitation, rather than a spontaneous attempt to re-initiate play after a natural interruption. Second, the instruction to both citizen scientists to smile and nod if the dog looked at them may be a limitation. We chose to have both citizen scientists smile and nod to stay consistent with other papers examining eye contact (e.g., Bray et al. 2020; Marshall-Pescini et al. 2013). However, this interaction could be seen as an encouragement to continue to engage, which could shed doubt on the interpretation of the behavior as being motivated solely by the desire to restart a joint commitment. However, our previous work did not include smiling and nodding and had similar results, so it does not seem to be the driving factor of re-engagement (Horschler et al. 2022). Third, there was high variability in the setting and the type of play elicited. Although we did not measure specific differences in play behavior, it is possible that differences between settings or play styles had some impact, but similarly of our results to those of Horschler and colleagues (2022) suggests that differences in play did not widely impact the results presented here.

Currently, work on dogs’ understanding of behavioral intentions is mixed, possibly due to the current research occurring in very specific, and often differing contexts (*for:* Marshall-Pescini et al. 2014; Schunemann et al. 2021; *against:* Moore et al. 2015), and further work could test how dogs understand the goals and intentions of others in varied contexts. For instance, future work could investigate other behavioral signatures of joint intentionality such as role-reversal, where the subject is able to alternate to their partners’ distinct role to maintain cooperative intent (Tomasello and Moll 2010). Excitingly, our results are closely aligned with those of analogous experimental work in a laboratory setting, supporting the idea that remote video studies of animal cognition, including experiments administered by citizen scientists, can yield valid and reliable results.

In sum, our study replicates and extends our previous work on joint intentionality to a new population of pet dogs. Our findings suggest that domestic dogs show preferential re-engagement of a former partner in response to an interrupted joint activity, a behavioral signature of joint intentionality.

## Supplementary Information

Below is the link to the electronic supplementary material.

Supplementary file1 (DOCX 30 KB)

## Data Availability

All data can be accessed by contacting the corresponding author directly.
