# Supplementary material for: Pet dogs (Canis familiaris) re-engage humans after joint activity
Source: Anim Cogn. 2023 Apr 13;26(4):1277–82. doi: 10.1007/s10071-023-01774-1 (PMC10345045; doi:10.1007/s10071-023-01774-1)
Supplement: Supplementary file 1 — Supplementary file1 (DOCX 30 KB) [file 10071_2023_1774_MOESM1_ESM.docx]

**Supplementary Materials**

**Main Analyses**

Models with continuous dependent measures (i.e., looking and physical contact) were fitted using the ‘lmer’ function with an identity link function and gaussian error distribution from the ‘lme4’ package (Bates et al., 2015). The model predicting toy offering was fitted using the ‘glmer’ function, specifying a log link function and poisson error distribution. For all models we assessed the effects of each term using the ‘Anova’ function from the ‘car’ package (Fox & Weisberg, 2019) to produce an analysis of deviance table using Type II Wald chi-square tests. For each model, we visually examined diagnostic plots to check for normality of residuals and linearity of fit. After examining diagnostic plots from models using untransformed outcome variables, we used a square-root transformation for the looking and physical contact variables to improve the normality of the residuals.

We also pre-registered a play score using the coding scheme from Horschler and colleagues (2022), which was from 1-3. We planned to do a subset analysis on only the trials in which dogs scored a 3, but coding by two coders (k = 0.92) revealed that 94% received the highest rating of playfulness (102/109 trials). Given the low variability, we did not report a subset analysis.

**Pre-registered Analyses**

After transforming the looking behavior and physical contact data into difference scores, we conducted one-sample t-tests to compare the difference scores to chance (0.5). Scores higher than chance would indicate that the behavior was mostly player-directed, and lower than chance would indicate that the behavior was mostly bystander-directed. We found that looking behavior was significantly player-directed (t(27) = 3.26, *p* = 0.003), but physical contact was not significantly different from chance (t(19) = -0.247, *p* = 0.8). This pattern is consistent with results from our main analyses.

**Exploratory Analyses**

In previous work by Horschler and colleagues (2022), all subjects were service-dogs-in-training, and all were either Labrador retrievers, golden retrievers, or Labrador x golden retriever crosses.  In the current study, there was high variability in breed and extent of training history. Due to this high variability, we examined a wider selection of re-engagement behaviors in an exploratory capacity. First was the number of instances of pawing at either person, defined as lifting the paw without a subsequent step or change in body position. Second, we coded licking either person, defined as the tongue leaving the dog’s mouth while they looked at a person (eyes or nose oriented above the person’s shoulders). Lastly, we coded nudging, which we defined as touching either person with the head or nose, while not holding the toy. Reliability for all of these behaviors was poor, but none of these behaviors occurred on more than 10% of trials (count: Pawing = 7, Licking = 9, Nudging = 9), and were thus excluded from further analyses.

We also fit linear mixed models on each of our dependent variables (looking, toy offering, and physical contact) including the person’s role and trial number as predictors, and the dog’s identity as a random effect. None of these models demonstrated a significant effect of trial, and did not have a large impact on the effect size of person’s role as compared to our main models. For this reason, and because we did not pre-register this analysis, we opted to include simpler models, without the trial term, in the main text.

|  | Touch | | Look | | Toy Offering | |
| --- | --- | --- | --- | --- | --- | --- |
|  | χ^2^(1) | P-value | χ^2^(1) | P-value | χ^2^(1) | P-Value |
| Person’s Role | 3.66 | 0.056 | 17.15 | <0.001*** | 4.82 | 0.028* |
| Trial | 0.35 | 0.56 | 0.93 | 0.33 | 3.1 | 0.078 |

Table S1. Demographic Data

| # | Familiarity of Person 1 (1+3) | Familiarity of Person 2 (2+4) | Dog Name | Sex | Age | Weight (lbs) | Breed |
| --- | --- | --- | --- | --- | --- | --- | --- |
| 1 | Very Well (owner) | Very well (owner) | Kyrie | Female | 9 | 58 | Golden Retriever |
| 2 | Very well | Very well | Clover | Female | 2 | 60 | Golden Retriever |
| 3 | Very well (owner) | Neighbor, every day | Bella | Female | 21 | 17 | Rat Terrier |
| 4 | Very well | Very well | Sadie | Female | 9 | 60 | Terrier Mix |
| 5 | Very well (owner) | Very well (owner) | Cookie | Female | 8 | 8 | Toy Poodle Mix |
| 6 | Very well (owner 3 months) | Very well (owner 3 months) | Echo | Female | 0 | 8.8 | Australian Shepherd |
| 7 | Very well (owner) | Very well (owner) | Bruce | Male | 8 | 45 | Collie Mix |
| 8 | Very well (owner) | Very well (owner) |  |  |  |  |  |
| 9 | Very well (owner) | Very well (Previously lived in the home) | Abby | Female | 5 | 60 | Labrador Retriever |
| 10 | Very well (owner) | Very well (owner) | Miles | Male | 3 | 75 | Golden Retriever |
| 11 | Very well (owner) | Very well (Previously lived in the home) | Louie | Male | 1 | 20 | wheaten terrier |
| 12 | Very well (owner) | Very well (owner) |  |  |  |  |  |
| 13 | Very well | Very well | Onyx | Female | 3 | 50 | Boxer Mix |
| 14 | Very well | Very well | Lolly | Female | 11 | 22 | Cavalier/Bichon Mix |
| 15 | Very well (owner) | Very well (owner) | Bruce | Male | 10 | 20 | Pug |
| 16 | Very well (owner) | Very well (brother) | Tucker | Male | 7 | 48 | Australian Shepherd Mix |
| 17 | Very well (owner) | Very well (owner) |  |  |  |  |  |
| 18 | Very well (owner) | Friend (sees dog twice a week) | Leo | Male | 3 | 42 | Australian Labradoodle |
| 19 | Very well | Very well | Rocco | Male | 2 | 34 | Mix |
| 20 | Very well (owner) | Very well (owner) | Stella | Female | 2 | 47 | Boxer/Hound Mix |
| 21 | Friend (known for 3 years) | Very well (owner) | Elliott | Male | 6 | 25 | Border Collie/Shi Tzu Mix |
| 22 | Friend (dog sitter, every day) | Very well (owner) | Sam | Male | 3 | 82 | Golden Retriever |
| 23 | Very well (owner) | Very well (owner) | Rocky | Male | 5 | 52 | Pitbull Mix |
| 24 | Very well | Very well | Rory | Female | 1 | 30 | Mix |
| 25 | Very well (owner) | Very well (owner) |  |  |  |  |  |
| 26 | Very well (owner) | Very well (owner) | Phantom | Male | 4 | 80 | German Shepherd Dog |
| 27 | Very well (owner) | Very well (owner) | Lando | Male | 4 | 16 | Chihuahua/Terrier Mix |
| 28 | Very well | Very well | Lyric | Female | 1 | 60 | Labrador retriever |
| 29 | Very well (owner) | Very well (owner) | Gus | Male | 3 | 16 | Cattle dog/ Boston Terrier Mix |
| 30 | Friend (every day for a month) | Very well (owner) | Bode | Male | 0 | 80 | German Shepherd Mix |
| 31 | Very well (owner) | Very well (owner) | Dallas | Male | 12 | 70 | Labrador Retriever |
| 32 | Very well (owner) | Very well (owner) | Max | Male | 1 | 20 | Pug |
| 33 | Very well (owner) | Very well (owner) |  |  |  |  |  |

Dogs are numbered in the order they were tested. Familiarity of dog to people (column one is for the player in trials 1 and 3 and column two is for the player in trials 2 and 4), dog’s name, sex, age at time of testing, weight, and breed. Data are missing for five dogs because the owners opted not to include their dog’s data. Due to the large variability in types of relationships dogs could have with people, owners were asked the open-ended question, “How well does your dog know both people?” and allowed to verbally respond however they wished. Every person answered “Very well”, but when they chose to elaborate on this categorization, that information is included in the familiarity of person columns. Only five dogs were reported as not living with both of the humans, and some participants did not explicitly specify the living situation.

**References**

Bates, D., Machler, M., Bolker, B. M., & Walker, S. C. (2015). Fitting linear mixed effects

models using lme4. Journal of Statistical Software, 67(1), 1e48. https://

doi.org/10.18637/jss.v067.i01

Fox, J., & Weisberg, S. (2019). An R companion to applied regression (3rd ed.). Thousand Oaks, CA: Sage.
